# Antifungal Activity of Menisporopsin A against Relevant Plant Pathogens

**DOI:** 10.3390/jof10060381

**Published:** 2024-05-27

**Authors:** Candelario Rodriguez, Masiel Barrios-Jaén, Luis C. Mejía, Marcelino Gutiérrez

**Affiliations:** 1 Centro de Biodiversidad y Descubrimiento de Drogas, Instituto de Investigaciones Científicas y Servicios de Alta Tecnología (INDICASAT AIP), Panama City 0843-01103, Panama; crodriguez@indicasat.org.pa (C.R.); mbarrios@indicasat.org.pa (M.B.-J.); 2 Smithsonian Tropical Research Institute, Ancón 0843-03092, Panama

**Keywords:** *Menisporopsis*, macrolactones, antifungal activity, menisporopsin A, endohytes, plant pathogens

## Abstract

Current agrochemicals used in crop farming mainly consist of synthetic compounds with harmful effects on the environment and human health. Crop-associated fungal endophytes, which play many ecological roles including defense against pathogens, represent a promising source for bioactive and ecologically safer molecules in agrochemical discovery. The methanolic extract of the endophyte *Menisporopsis* sp. LCM 1078 was evaluated in vitro against the plant pathogens *Boeremia exigua*, *Calonectria variabilis*, *Colletotrichum theobromicola*, *Colletotrichum tropicale*, and *Mycena cytricolor*. Bioassay-guided isolation using chromatographic techniques followed by detailed chemical characterization by NMR and mass spectrometry led to the identification of menisporopsin A, which showed inhibitory activity in a dose-dependent manner against the five fungal pathogens including an endophytic strain (*Colletotrichum tropicale*), with MIC values in the range of 0.63–10.0 μg/mL showing a potency equivalent to the broadly employed agrochemical mancozeb.

## 1. Introduction

Since ancient times, with the first attempts at agriculture, the life of commercially relevant plants has been affected by pathogens such as bacteria, fungi, and viruses, as well as pests like insects and worms [1]. According to the food and agriculture organization (FAO), the economic losses caused only by pests reach up to 220 billion per year, affecting the food security, income, and lifestyle of the people depending on these items to survive [2]. Phytopathogenic fungi represent the most dispersed causal agents of plant diseases worldwide [1]. These microorganisms may cause diseases in several crop plants; for example, *Boeremia exigua* affects beans, fruits, potatoes, and woody plants [3]; *Calonectria variabilis* causes leaf spots on medicinal plants and *Theobroma grandiflorum* [4]; *Colletotrichum theobromicola* is known to produce anthracnose on cherries, olives, and leaf spots in ornamental and odorous plants [5]; *Colletotrichum tropicale* has been reported as an endophyte in many plants but also as a pathogen infecting palms and many native plants from tropical regions [6,7]; and *Mycena citricolor* represents the most severe disease on *Coffea arabica* after the coffee leaf rust [8]. Crop losses attributed to diseases caused by fungal pathogens worldwide range between 10% and 23% per year at the harvest stage, while losses of around 20% at the post-harvest stage are accounted for [9]. Furthermore, climate change is visualized as influencing the spreading of pathogens to the half of the earth inhabited by their hosts by the middle of this century [1,2]. Hence, the discovery of new and ecologically safe agrochemicals is imperative.

Among the practices employed to handle fungal diseases on crops, there are cultural, biological, and chemical controls. Traditional crop management includes agroforestry, which integrates a series of practices such as the removal of infected tissues like leaves and branches [10]. Plants infected with *Boeremia exigua* are treated by pruning superior stems, which allows for air flow at basal stems, thus reducing the diseases [11]. Biological controls have been a sustainable option to control fungal diseases. Some *Colletotrichum* diseases are treated using *Trichoderma* species or *Bacillus subtilis* [12].

Current agrochemicals applied in crop farms consist of inorganic formulations or organic compounds produced by chemical synthesis [13,14]. The control of the *Calonectria* species is carried out by application of azoles and analogs, which sometimes do not meet with government approval [15]. The *Colletotrichum* species may be managed by removing dead leaves and a further spraying of tebuconazole [16]. Fungicides based on cupper formulations have been suggested to be applied for the control of *Mycena citricolor* in Central America [17]. Furthermore, in fruits and vegetables, the employment of agrochemicals needs to continue even weeks post-harvesting [18]. Improper applications of these substances can cause environmental pollution in addition to the occurrence of residual contaminants on final crop products. In addition, the dysregulation of endophytic microbiota that naturally occurs on commercially relevant plants after the application of agrochemicals has been ignored [19].

Endophytic microbes include bacteria and fungi that live inside plants without causing damage or harmful effects. The relationship that forms the endophytes and their host is governed by symbiosis. The microbial partner of this consortia plays many ecological roles, including defenses of their host against pathogens through the production of secondary metabolites. On the other hand, the host provides polymeric substrates such as polysaccharides and proteins that serve as food for microbes’ growth [20].

Endophytes, which are present in most plant species, represent a safer alternative to the use of agrochemicals that are harmful to the environment and to human beings [21,22]. In particular, natural products produced by endophytic fungi possess a great biotechnological potential in crop protection [22,23]. The use of fungal endophytes and their secondary metabolites as natural protective agents represents a promising resource for more active and safer compounds in agrochemical discovery [24].

The macrocyclic polylactone menisporopsin A was first isolated from the endophytic fungus *Meniporopsis theobromae* BCC 3975 associated with *Theobroma cacao* seeds [25]. This polyketide-type metabolite has been the focus of much attention because it showed antiparasitic activity against the multidrug-resistant strain K1 of *Plasmodium falciparum* and antibacterial activity against *Mycobacterium tuberculosis* (strain H37Ra), as well as anticancer activity against BC-1 (breast cancer) and KB (nasopharyngeal carcinoma) cells. It is intriguing that although antifungal activity is known for many macrolactones, menisporopsin A showed no activity against *Candida albicans*, and bioactivity against other fungi has not been reported so far [25].

As part of our program to search for natural products with agrochemical and biomedical applications, we have created a collection of fungal endophytes with about 6000 isolates. Of these, about 3500 have been obtained from *Coffea arabica* plants, and a plethora of microbial interactions have been observed, including inhibition against several phytopathogens. Bioassay-guided isolation of the bioactive crude extract obtained from the endophyte *Menisporopsis* sp. LCM 1078 led to the isolation and identification of menisporopsin A. Herein, we investigated the potential of menisporopsin A as an agrochemical for crop protection against plant pathogenic fungi of high relevance in agriculture and compared its biological activity with that of mancozeb, a commercial chemical with a wide use in agriculture.

## 2. Materials and Methods

This work describes the isolation, chemical structure, and novel antifungal activity of menisporopsin A produced by plant endophyte *Menisporopsis* sp. LCM 1078 associated with *Coffea arabica* leaves.

### 2.1. Isolation and Identification of the Endophyte Menisporopsis sp. LCM 1078

The endophyte was isolated from leaves of *Coffea arabica* collected in the Province of Chiriquí, Panama. The leaf source of this isolate was processed by performing leaf surface sterilization following methods described in Mejía et al. 2008 [24], followed by placing pieces of clean leaves in 2% malt extract agar. Hyphal tip of the fungus was transferred to potato dextrose agar, and voucher specimens were made from there for long-term storage in the microbial collection of INDICASAT. *Menisporopsis* sp. strain LCM 1078 was identified based on molecular methods and substantiated by the identities of the chemical compounds produced by this fungus that are characteristic of this genus and here reported as antifungals. The ITS sequence was generated following DNA extraction, PCR, and sequencing methods as we previously described [26]. The ITS sequence of the fungus was compared to DNA sequences available in Genbank using BLAST and a % sequence identity > 97% in comparison with reference strains was considered sufficient for genus assignment. The ITS sequence of this fungus is already publicly available in Genbank under accession number PP531610.

### 2.2. Culture Conditions and Chemical Extraction of the Menisporopsis sp. LCM 1078

Seed fungus was inoculated in potato dextrose agar (PDA). Then, 1-cm^2^ plugs were placed at the center of 20 mL petri dishes containing PDA and incubated for 32 days at 25 ± 2 °C. After incubation, fifty-eight plates were accounted to be without contaminants and were freeze-dried for 72 h. Dried mycelia were crushed and extracted twice with 500 mL of solvent. Methanol was selected as solvent due to its ability to extract a broad number of secondary metabolites [27]. The macerated material was then concentrated to dryness to produce 827 mg of crude extract.

### 2.3. Nuclear Magnetic Resonance, Mass Spectrometry, and High-Performance Liquid Chromatography Analysis

Nuclear magnetic resonance (NMR) experiments were carried out at 25 °C on a JNM-ECZ500R/S1 Jeol Fourier transform spectrometer with a field strength of 11.74 T and equipped with a 5 mm TH tunable probe (Jeol, Peabody, MA, USA). NMR spectra were referenced based on the residual solvent signal of acetone-d (δ_H_ 2.05 and δ_C_ 29.84) or methanol-d (δ_H_ 3.31 and δ_C_ 49.00) [28]. NMR data were processed with the MestReNova software version 12.0.3-21384 (© Mestre-lab Research S.L., Santiago de Compostela, Spain). For the molecular weight determination, samples were diluted in methanol LC-MS grade (LiChrosolv) at 0.1 mg/mL and directly infused into the mass spectrometer. Analysis was performed in an XEVO^®^ TQD spectrometer coupled to an electrospray ionization (ESI) source (Waters Corporation, Milford, MA, USA). MS spectra were obtained in positive electrospray ionization mode in the range of 50 to 2500 mass-to-charge ratio (*m*/*z*). The capillary voltage was set at 4500 V, and MSMS analysis was performed by setting the collision cell at 20 V. Nitrogen was used as the nebulizer gas at 2.0 bar, 200 °C, and 9.0 L/min. Reverse-phase high-performance liquid chromatography was carried out on an Agilent 1100 HPLC equipped with a diode array detector 1200 series (Agilent, Santa Clara, CA, USA) and a reverse phase column Fusion C18, 250 × 10 mm, 4 μm, 80 Å (Phenomenex^®^, Torrance, CA, USA).

### 2.4. Isolation and Chemical Characterization

The crude extract was pre-fractionated by vacuum solid phase extraction (VSPE) using a visiprep SPE vacuum manifold (Supelco, Bellefonte, PA, USA) equipped with C-18 cartridges (Supelco, Bellefonte, PA, USA). A stepwise gradient of 20, 40, 60, 80, and 100% of methanol in water, eluted at 8 mL/min, was employed to obtain five fractions A-E. Samples were submitted to biological assays, and the active fraction D was subjected to reverse-phase high-performance liquid chromatography (HPLC), employing a semi-preparative column. Fraction D was dried and suspended in methanol to obtain a stock concentration of 40 µg/µL. Injections of 50 μL (2 mg) were carried out, and elution was set at a flow rate of 2 mL/min. The solvent system consisted of water (solvent A) and methanol (solvent B). Elution was conducted using a gradient from 50% to 100% of solvent B in solvent A for 80 min. Peaks collected were dried under reduced pressure at 30 °C. Structural elucidation of bioactive principles was carried out by mass spectrometry and nuclear magnetic resonance spectroscopy.

### 2.5. Compound Characterization Data

Menisporopsin A is a yellow solid for ^1^H and ^13^C NMR spectroscopic data see Supplementary Information file (Figures S1–S6). Measured ESI-MS *m*/*z* 821.2650 [M + Na]^+^ (calculated for C_40_H_46_NaO_17_, error 2.8 ppm). Additionally, cleavage sites for MS/MS fragmentation of menisporopsin A are shown in Figure S7.

### 2.6. Plant Pathogens

The following plant pathogenic strains of fungi used in our assays were available from the culture collection of INDICASAT: *Boeremia exigua* DE 86 isolated from *Coffea arabica*, *Calonectria variabilis* LCM 1067 isolated from *Anacardium occidentale*, *Colletotrichum theobromicola* ER 0811 isolated from *Theobroma cacao*, *Mycena citricolor* MC 01 isolated from *Coffea arabica. Colletotrichum tropicale* 5101, isolated as an endophyte from *Theobroma cacao*, was also included in the assay.

### 2.7. Antifungal Activity

In vitro antifungal activity was evaluated on solid media employing the poisoned food bioassay method. Samples were dissolved in dimethyl-sulfoxide (DMSO) and added to potato dextrose agar (PDA) medium. The final concentration of DMSO in PDA was 0.5% *v*/*v*. The temperature was kept at 40 °C, and 5 mL of this bioprospecting medium was transferred to 6 cm diameter Petri dishes. After, a plug of the pathogen was taken with a 5 mm hole puncher and inoculated at the center of the plates. Treatments were carried out at 25 °C [29].

Bioactivity was determined as growth inhibition percentages. Mycelial growth of fungal pathogens was measured by the cross-bracket method, and growth inhibition percentages were calculated as GI %: (1 − T/c) × 100, where c corresponds to the radial growth of pathogen in the negative control and T is equal to the radial growth of pathogen under treatments [30].

For the screening, mancozeb was dissolved in DMSO, added to a final concentration of 100 µg/mL, and used as a positive control for growth inhibition. As negative control, we employed DMSO at 0.5% *v*/*v*. Plant pathogens did not show differences in growth between plates with PDA and plates with DMSO at 0.5% *v*/*v* in PDA (Figure S8). SPE fractions (A–E) were dissolved in DMSO (final concentration 0.5% *v*/*v*) and evaluated at 100 µg/mL. Minimal inhibitory concentration (MIC) was defined as the sample’s lowest concentration, which shows visible growth inhibition after incubation by 48 h [31]. MIC of mancozeb and menisporopsin A were determined at a series of two-fold concentrations. Effective concentration that inhibits 50% of the mycelial growth (EC_50_) was determined graphically. Aqueous solutions of mancozeb and menisporopsin A were evaluated on leaves of *Coffea arabica* and no issues with mobility were observed (unpublished results).

### 2.8. Statistical Analysis

Inhibition of plant pathogens is shown as mean ± standard error of the mean, and each treatment was replicated three times. The analysis of variance was carried out employing the Shapiro–Wilk normality and the non-constant variance score tests. Data analysis for ANOVA were managed by using the R statistical package version v.4.3.0 (R Foundation for Statistical Computing, Viena, Austria) [32].

## 3. Results

**Figure 1:**
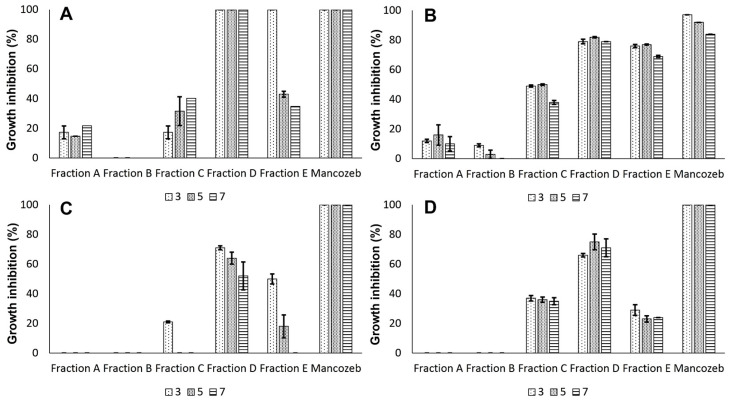
Bioguided screening is presented as mean values with standard error of the mean. Column fractions evaluated at 3, 5, and 7 days. Mancozeb corresponds to the positive control. Fungal pathogens correspond to *Boeremia exigua* DE 86 (**A**), *Calonectria variabilis* LCM 1067 (**B**), *Colletotrichum theobromicola* ER 0811 (**C**), and *Mycena citricolor* MC 01 (**D**). Growth inhibition was calculated in comparison with the negative control. No statistical difference was found between days of treatments (*p*: 0.183). Bioactivity of fractions displays statistical difference (*p* < 0.001).

**Figure 2:**
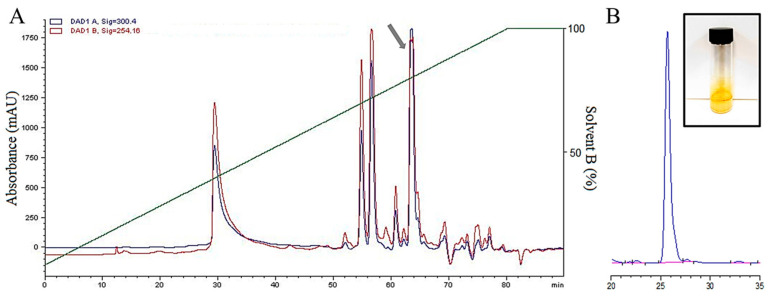
HPLC profile for the separation of the bioactive fraction D, the peak corresponding to menisporopsin A is shown with a gray arrow (**A**). Purity analysis by HPLC-UV at 254 nm of menisporopsin A. HPLC chromatographic conditions: semi-preparative Fusion C18 column. Mobile phase: solvent A: water and solvent B: methanol. Gradient system of solvent B: 75–100% by 40 min. Retention time for menisporopsin A was 25.66 min (**B**).

The methanolic extract of the fungus *Menisporopsis* sp. LCM 1078 was partitioned by C18 solid phase extraction (SPE) in five fractions (A–E), producing 386.1 mg for fraction A, 170.5 mg for fraction B, 51.8 mg for fraction C, 72.1 mg for fraction D and 97.2 mg for fraction E. The fractions A and B did not present activity after 48 h of treatment (Figure S9). On the other hand, fraction C showed mild inhibition but kept bioactivity against *C. variabilis* LCM 1067 even after seven days of treatment. Remarkably, it was found that *B. exigua* DE 86, *C. variabilis* LCM 1067, *C. theobromicola* ER 0811, and *M. citricolor* MC 01 were all inhibited by the fraction D. In particular, inhibition of fraction D against *Boeremia exigua* DE 86 was equivalent to the effect produced by mancozeb, which showed 100% inhibition with almost all pathogens at 100 μg/mL (Figure 1). The fraction E showed inhibition against *C. variabilis* LCM 1067; however, the inhibition of *B. exigua* DE 86, *C. theobromicola* ER 0811, and *M. citricolor* MC 01 decreased after 48 h of treatment (Figure 1). Therefore, fraction D was subjected to purification by semipreparative HPLC. The chromatographic profile showed five main peaks (Figure 2A). The peak corresponding to menisporopsin A (shown with a gray arrow) displayed antifungal activity; hence, it was analyzed by spectral purity at 254 nm (Figure 2B). The recovery amount of menisporopsin A was determined as 22.9 mg.

**Figure 3:**
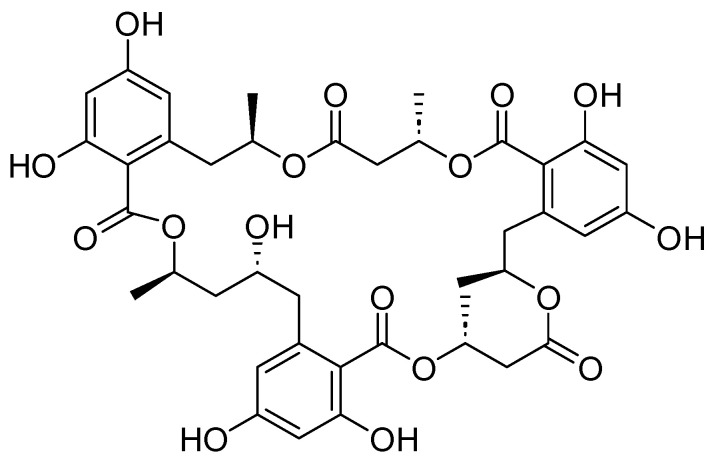
Chemical structure of menisporopsin A.

Detailed chemical characterization of menisporopsin A (Figure 3) was performed by nuclear magnetic resonance (NMR) and mass spectrometry (MS). All spectroscopic data observed experimentally for the isolated menisporopsin A were in agreement with those reported in the literature [25]. Attempts to dissolve menisporopsin A in chloroform were unsuccessful; however, chemical shifts in methanol-d are reported (Table S1).

**Figure 4:**
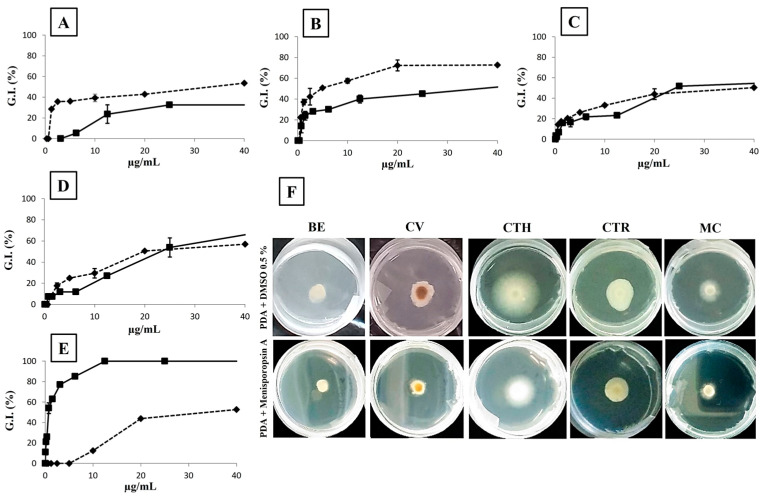
Growth Inhibition (G.I. %). Dose-response curve for the antifungal activity of menisporopsin A (dashed line) and mancozeb (solid line) against (**A**) *Boeremia exigua*, (**B**) *Calonectria variabilis*, (**C**) *Colletotrichum theobromicola*, (**D**) *Colletotrichum tropicale*, and (**E**) *Mycena citricolor*. (**F**) Upper panel: Growth of plant pathogens on negative control. Lower panel: Evaluation of menisporopsin A at 40 µg/mL against five plant pathogens after 48 h. DMSO was mixed with PDA and employed as negative control. Fungi are coded as BE (*Boeremia exigua*), CV (*Calonectria variabilis*), CTH (*Colletotrichum theobromicola*), CTR (*Colletotrichum tropicale*), and MC (*Mycena citricolor*). Growth inhibition was calculated in comparison with the negative control. Statistical difference between doses (*p* < 0.001).

Menisporopsin A showed inhibitory activity against *B. exigua* DE 86, *C. variabilis* LCM 1067, *C. theobromicola* ER 0811, *C. tropicale* 5101, and *M. citricolor* MC 01 (Figure 4). In vitro evaluation showed EC_50_ values of 35 µg/mL against *B. exigua* DE 86, *M. citricolor* MC 01, and *C. theobromicola* ER 0811. On the other hand, for *C. variabilis* and *C. tropicale*, the EC_50_ obtained were 5 µg/mL and 20 µg/mL, respectively. Mancozeb displayed an EC_50_ of 25 µg/mL for *Colletotrichum* strains, while EC_50_ values of 35 µg/mL and 1 µg/mL were obtained for *Calonectria variabilis* LCM 1067 and *Mycena citricolor* MC 01, respectively. In the case of *Boeremia exigua* DE 86, mancozeb showed an EC_50_ above the tested concentrations and hence was calculated as >40 µg/mL. The MIC determination for menisporopsin A revealed values of 0.62 µg/mL against *C. variabilis* LCM 1067 and *C. theobromicola* ER 0811 and 1.25 µg/mL against *B. exigua* DE 86 and *C. tropicale* 5101. On the other hand, the MIC value of menisporopsin A against *M. citricolor* MC 01 was 10 µg/mL (Figure S10). The bioactivity of mancozeb against the five strains evaluated ranged from 0.09 to 6.25 µg/mL (Table 1). Growth inhibition of plant pathogens at the maximum concentration tested of menisporopsin A is shown in Figure 4F. After seven days of treatment with menisporopsin A, an agar plug with mycelia from the edge of the colony of the evaluated strains was seeded on PDA, and growth was observed (Figure S11).

## 4. Discussion

The antifungal activity of menisporopsin A showed a dose-response curve against *B. exigua* DE 86, *C. variabilis* LCM 1067, *C. theobromicola* ER 0811, *C. tropicale* 5101 and *M. citricolor* MC 01 (Figure 4). This dose-dependent behavior and the fact that the strains evaluated grew after being treated for seven days suggests a possible mechanism of fungistatic action rather than fungicidal activity. Some macrolactones have shown antifungal activity by inhibiting the cell wall biosynthesis of *Neurospora crassa* [33]. Further investigations that need to be conducted on menisporopsin A as a potential agrochemical would involve the determination of the antifungal mechanism of action and phytotoxicity experiments. Macrolatones isolated from the mangrove-associated endophytic fungi *Lasiodiplodia theobromae* displayed no phytotoxicity on leaves of *Digitaria ciliaris*; however, after the opening of the ring, significant toxicity was observed [34].

The MIC value allows us to measure the sensibility threshold that a microbe shows against a substance and is determined as the lowest amount of bioactive substance needed to cause visible growth inhibition [31]. In this regard, menisporopsin A showed a potency comparable to mancozeb by inhibiting *Calonectria* and *Colletotrichum* strains. On the other hand, its potency was slightly superior against *B. exigua* DE 86 and a hundred-fold less active than mancozeb against the basidiomycota *M. citricolor* MC 01 (Figure 4, Table 1). The results observed in Figure 4A–D at multiple concentrations are consistent with the observed in Figure 4F at the single concentration of 40 µg/mL. According to some studies, the effective concentration is also used to estimate antifungal potency [35]. Some studies have reported the bioactivity of mancozeb employing the same methodology, based on treatment by poisoned food bioassay. After 25 days of incubation, mancozeb showed an EC_85_ of 4.91 µg/mL against the mycelial growth of *Calonectria pseudonoviculata* [36]; this is comparable with the EC_50_ value obtained from menisporopsin A against *Calonetria variabilis* LCM 1067. Serial dilutions of mancozeb at 500, 1000, and 1500 µg/mL displayed 61.90%, 78.91%, and 90.48% of inhibition against *Boeremia exigua*, respectively, after 10 days of incubation [37]. In this study, we obtained similar EC_50_ values for *Colletotrichum* species (20–35 µg/mL) for menisporopsin A and mancozeb. However, in a previous report, it was found that mancozeb at 250 µg/mL displayed 31.11% inhibition against *Colletotrichum gloeosporioides* after 8 days of treatment [38]. After 3 days of treatment, mancozeb displayed 36.9% mycelial inhibition at 30 µg/mL against *Mycena citricolor* in the same range of the EC_50_ for menisporopsin A that we observed in this study [39]. The difference in potencies between positive controls may be the quality of the strains and possible resistance acquired by wild-type strains. Furthermore, it is intriguing that previously, menisporopsin A showed no antifungal activity at 20 µg/mL against *Candida albicans*, a unicellular ascomycota, and no antifungal activity against other fungi has been reported [25]. In the search for potential agrochemicals, differential bioactivity may warrant interaction with specific and important targets.

In the present work, menisporopsin A was purified from the fungus *Menisporopsis* sp. LCM 1078, which was isolated as an endophyte from *Coffea arabica*’s leaves. Endophytic fungi have coevolved with plants and provided them with natural tools for healthy growth and life. The research on ecological interactions of this system is still ongoing; however, there are four main roles described [40]. Fungal endophytes are involved in the nutrient uptake mechanism. Species of the genus *Aspergillus*, *Cochliobolus*, *Ophiosphaerella*, and *Setosphaeria* can improve calcium sequestration in plants [41]. Plants treated with endophytes have shown tolerance to abiotic stress. The fungi *Phoma glomerata* and *Penicillium* sp. were able to induce resistance against salinity and drought events as compared to uninoculated plants [42]. Fungal endophytes are applied as biopesticides in the crop industry. *Beuveria bassiana* produces a degrading enzyme to penetrate insects and deliver mycotoxins [43]. Fungal phytopathogens can be managed by treatment with endophytic fungi from the genera *Acremonium*, *Aspergillus*, *Epichloe*, *Neotyphodium*, and *Trichoderma* [44]. In general, the mechanisms reported to be involved in the inhibition of fungal pathogens are the upregulation of the phytohormones, increase in metabolic and photosynthetic rates, and production of bioactive secondary metabolites [45].

Mancozeb is an organometallic compound of the thiocarbamate family applied as a fungicide agent worldwide in the protection of several crops, mainly fruits and vegetables, at doses around 5 mg/mL [46,47]. However, its environmental effects and approval for continuous use is a topic of current debate [13]. Mancozeb inhibits fungi through multiple pathways, including the generation of radical oxygen species and disruption of the metabolism of lipids [47]. Some of the ecological concerns during the use of mancozeb include toxic effects on animals, residual concentration in final products, and effects on the health of soil microbiota. Moreover, studies on birds showed disruption in thyroid homeostasis and reproduction after 30 days of exposure to this agrochemical at a low dose (0.5% LD50) [48]. Also, mancozeb causes severe disturbances such as epidermal necrolysis in humans [49]. An analysis of fruits and vegetables by LCMS revealed amounts exceeding the maximum residue level set by the European Union for human consumption [50]. On the other hand, it has been proven that the balance of bacteria and fungi in the soil of farms can be modified by mancozeb at agricultural doses (2–10 kg per hectare). While fungi are less affected, denitrifying bacteria and diazotrophs were significantly decreased [51].

Overuse of synthetic/inorganic fungicides has provoked pathogenic resistance, highlighting the need for novel agrochemical scaffolds [52]. The pathogens *Calonectria pauciramosa* and *Calonectria polizzii* display higher effective concentration values against the chlorinated azole prochloraz in ornamental nursery plants. EC_50_ ranges were increased from 5.1–10 to 10.1–25 µg/mL [53]. The *Colletotrichum* species shows a great incidence of fungicide resistance against strobilurin, thiophanate-methyl, and azoxystrobin [54]. Genomic analysis has allowed the determination of single mutations in amino acid residues in tubulin, mitochondrial cytochrome B, and sterol 14α-demethylase as responsible for the *Colletotrichum* fungicide resistance. These mutations are related to the uproper binding between fungal proteins and agrochemicals [55]. On the other hand, since it was launched in 1948, there have not been reports on the resistance of fungal phytopathogens against mancozeb in spite of being a fungicide applied to several crops [47].

Among the main challenges to overcome in agrochemical discovery are finding active ingredients that meet a series of features such as high potency, low cost, and environmental friendliness [56]. Nature can be visualized as the best synthetic chemist due to the high complexity and diversity observed in the carbon skeletons of natural products with a wide range of bioactivities [57]. In this study, we isolated the macrolactone menisporopsin A from the *Coffea arabica*’s endophytic fungus *Menisporopsis* sp. LCM 1078. Menisporopsin A is biosynthesized through the polyketide pathway with the participation of an additional non-ribosomal peptide synthetase-like enzyme required for esterification and lactonization steps [58]. The optimal culture conditions of menisporopsin A were investigated. The yield was increased from 348 mg/L to 889 mg/L, and cultivation time was reduced from 28 to 4 days. The culture conditions consisted of an initial pH of 8, agitation of 200 revolutions per minute, 1% of fructose as a carbon source, and 25% of meat extract as a nitrogen source [59].

## 5. Conclusions

This study represents the first report on the broad antifungal activity of menisporopsin A isolated from the endophyte *Menisporopsis* sp. LCM 1078. This compound showed a good antifungal profile against four plant pathogens, *B. exigua*, *C. variabilis*, *C. theobromicola*, and *M. citricolor*, and against *C. tropicale*, which has been reported as an endophyte and as a pathogen. The observed antifungal profile of menisporopsin A on these fungi of agricultural importance had a potency comparable to the commercial agrochemical mancozeb. Menisporopsin A seems to act through a fungistatic action rather than a fungicidal mechanism of action. This work confirms the general idea that endophytic fungi associated with crops are an extraordinary source of bioactive and environmentally safe molecules for agrochemical discovery and development.

## Figures and Tables

**Table 1:** MIC values for menisporopsin A and mancozeb against phytopathogens.

Plant Pathogen	MIC (µg/mL) ^1^		EC_50_ (µg/mL)	
	Mancozeb	Menisporopsin A	Mancozeb	Menisporopsin A
*Boeremia exigua* DE 86	6.25	1.25	>40	35
*Calonectria variabilis* LCM 1067	0.78	0.62	35	5
*Colletotrichum theobromicola* ER 0811	0.19	0.62	25	35
*Colletotrichum tropicale* 5101	0.78 ± 0.11	1.25	25	20
*Mycena citricolor* MC 01	0.09 ± 2.7	10.00	1	35

^1^ MIC are reported as mean values ± standard error of the mean (n = 3).

## Data Availability

All data are available in the main text and Supplementary Materials.

## Supplementary Materials

The following supporting information can be downloaded at https://www.mdpi.com/article/10.3390/jof10060381/s1, Figure S1: ^1^H NMR spectra of menisporopsin A at 500 MHz. Figure S2: ^13^C NMR spectra of menisporopsin A at 125 MHz. Figure S3: ^13^C NMR spectral edition of menisporopsin A at 125 MHz. Figure S4: ^1^H-^13^C (*J*^1^) HSQC spectra of menisporopsin A at 500 MHz. Figure S5: ^1^H-^1^H (*J*^3^) COSY spectra of menisporopsin A at 500 MHz. Figure S6: ^1^H-^13^C (*J*^3,4^) HMBC spectra of menisporopsin A at 500 MHz. Figure S7: MSMS spectrum and cleavage sites for fragmentation of menisporopsin A. Figure S8: Growth of plant pathogens by the poisoned food method after 3 days at 25 °C on petri dishes containing potato dextrose agar (PDA) and PDA mixed with Dimethyl-sulfoxide at a final concentration of 0.5% *v*/*v*. Figure S9: Antifungal activity against plant pathogens by the poisoned food method at 48 h of column fractions from the endophytic fungus *Menisporopsis* sp. LCM 1078. Figure S10: Antifungal activity against plant pathogens by the poisoned food method at 48 h of menisporopsin A. Figure S11: Treatment by the poisoned food method with menisporopsin A at 50 µg/mL by 7 days (A) and growth on PDA after 48 h of a plug taken from pathogen that were under treatment with menisporopsin A. Table S1: ^13^C NMR chemical shifts of menisporopsin A. Reference [25] is cited in the supplementary materials.
