# Supplementary material for: Antifungal Activity of Menisporopsin A against Relevant Plant Pathogens
Source: J Fungi (Basel). 2024 May 27;10(6):381. doi: 10.3390/jof10060381 (PMC11204650; doi:10.3390/jof10060381)
Supplement: Supplementary file 1 [file jof-10-00381-s001.zip › jof-2996402-supplementary.pdf]

Antifungal activity of menisporopsin A against plant pathogens

Candelario Rodriguez [1], Masiel Barrios-Jaén [1], Luis C. Mejía [1,2],* and Marcelino Gutiérrez [1],*

[1]Centro de Biodiversidad y Descubrimiento de Drogas, Instituto de Investigaciones Científicas y Servicios de Alta Tecnología (INDICASAT AIP), Panamá 0843-01103, Panama; crodriguez@indicasat.org.pa (C.R.); mbarrios@indicasat.org.pa (M.B)

[2]Smithsonian Tropical Research Institute, Ancón 0843-03092, Panamá

*Correspondence: lmejia@indicasat.org.pa (L.C.M); mgutierrez@indicasat.org.pa (M.G.)

Supporting Information

Content

Figure S1. 1H NMR spectra of menisporopsin A at 500 MHz.

Figure S2. ^{13}C NMR spectra of menisporopsin A at 125 MHz.

Figure S3. ^{13}C NMR spectral edition of menisporopsin A at 125 MHz.

Figure S4. ^{1}H-^{13}C (J^1) HSQC spectra of menisporopsin A at 500 MHz.

Figure S5. ^{1}H-^{1}H (J^3) COSY spectra of menisporopsin A at 500 MHz.

Figure S6. ^{1}H-^{13}C ($J^{3,4}$) HMBC spectra of menisporopsin A at 500 MHz.

Figure S7. MS/MS spectrum and cleavage sites for the fragmentation of menisporopsin A.

Figure S8. Growth of plant pathogens by the poisoned food method after 3 days at 25 °C on petri dishes containing potato dextrose agar (PDA) and PDA mixed with Dimethyl-sulfoxide at a final concentration of 0.5 % V/V.

Figure S9. Antifungal activity against plant pathogens by the poisoned food method at 48 hours of column fractions from the endophytic fungus *Menisporopsis* sp. LCM 1078.

Figure S10. Antifungal activity against plant pathogens by the poisoned food method at 48 hours of menisporopsin A.

Figure S11. Treatment by the poisoned food method with menisporopsin A at 50 μg/mL by 7 days (A) and growth on PDA after 48 hours of a plug taken from pathogen that were under treatment with menisporopsin A.

Table S1. ^{13}C NMR chemical shifts in acetone-d and methanol-d of menisporopsin A.

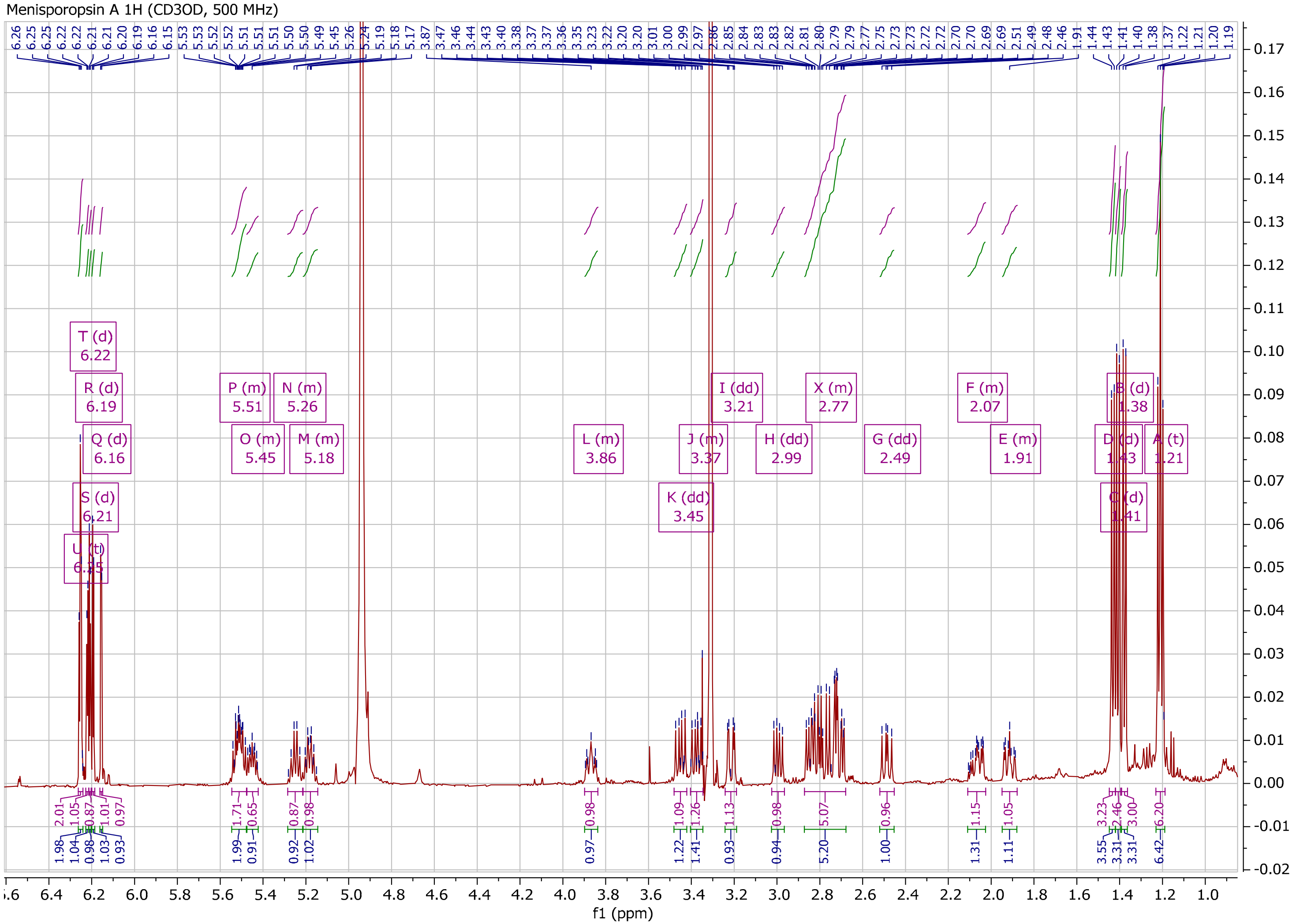

Figure S1. 1H NMR spectra of menisporopsin A at 500 MHz.

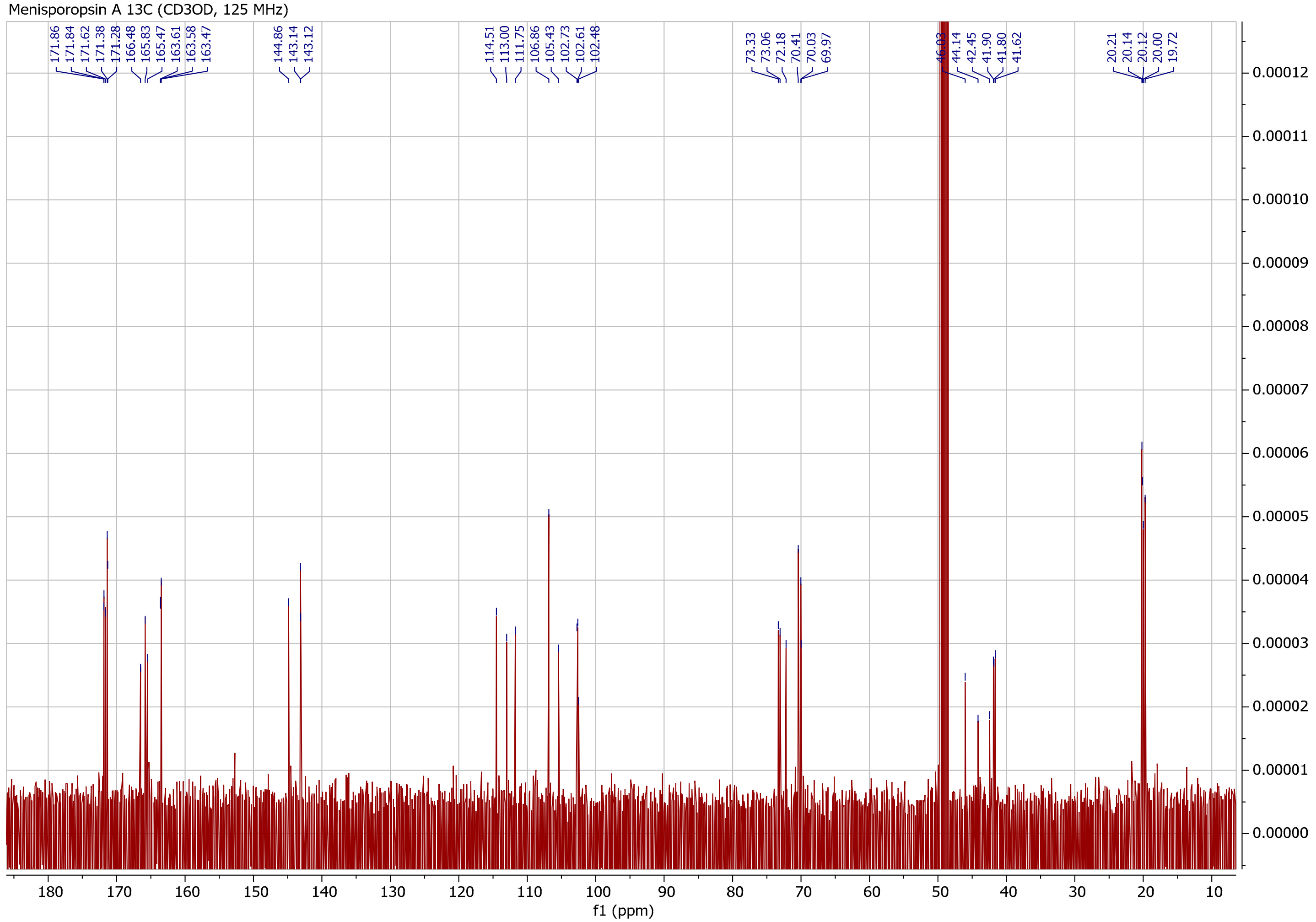

Figure S2. ^{13}C NMR spectra of menisporopsin A at 125 MHz.

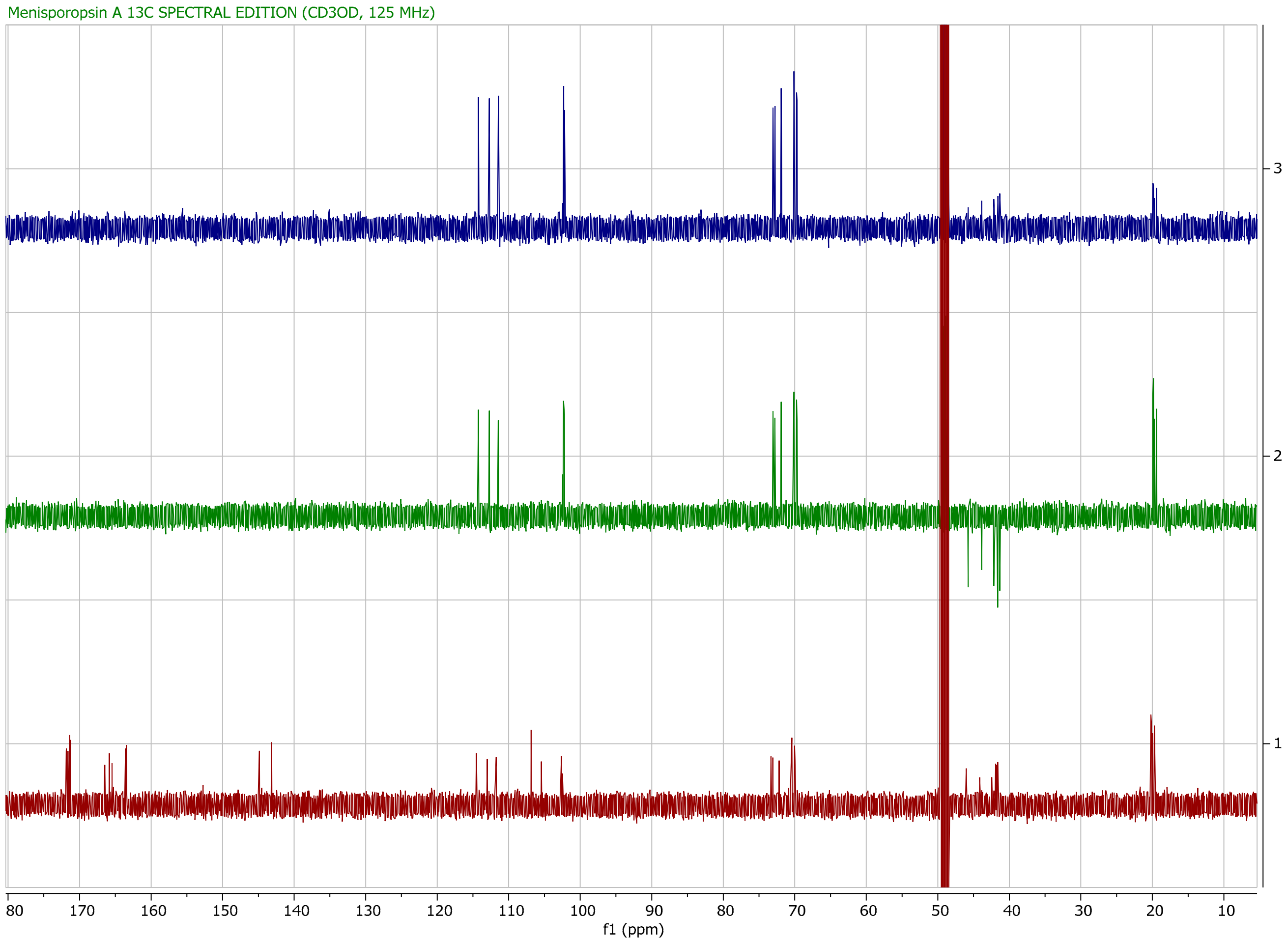

Figure S3. ^{13}C NMR spectral edition of menisporopsin A at 125 MHz.

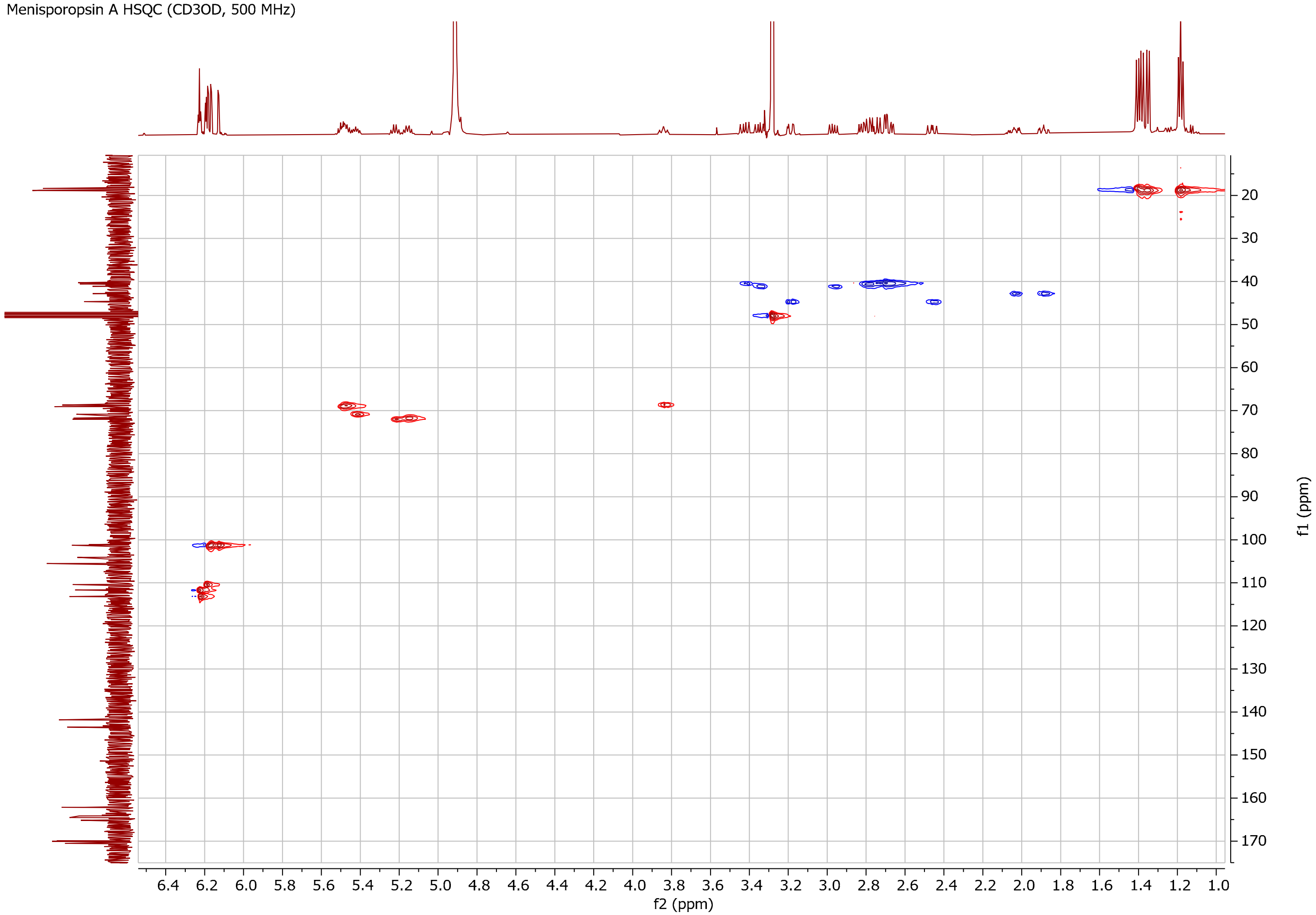

Figure S4. ^{1}H-^{13}C (J^1) HSQC spectra of menisporopsin A at 500 MHz.

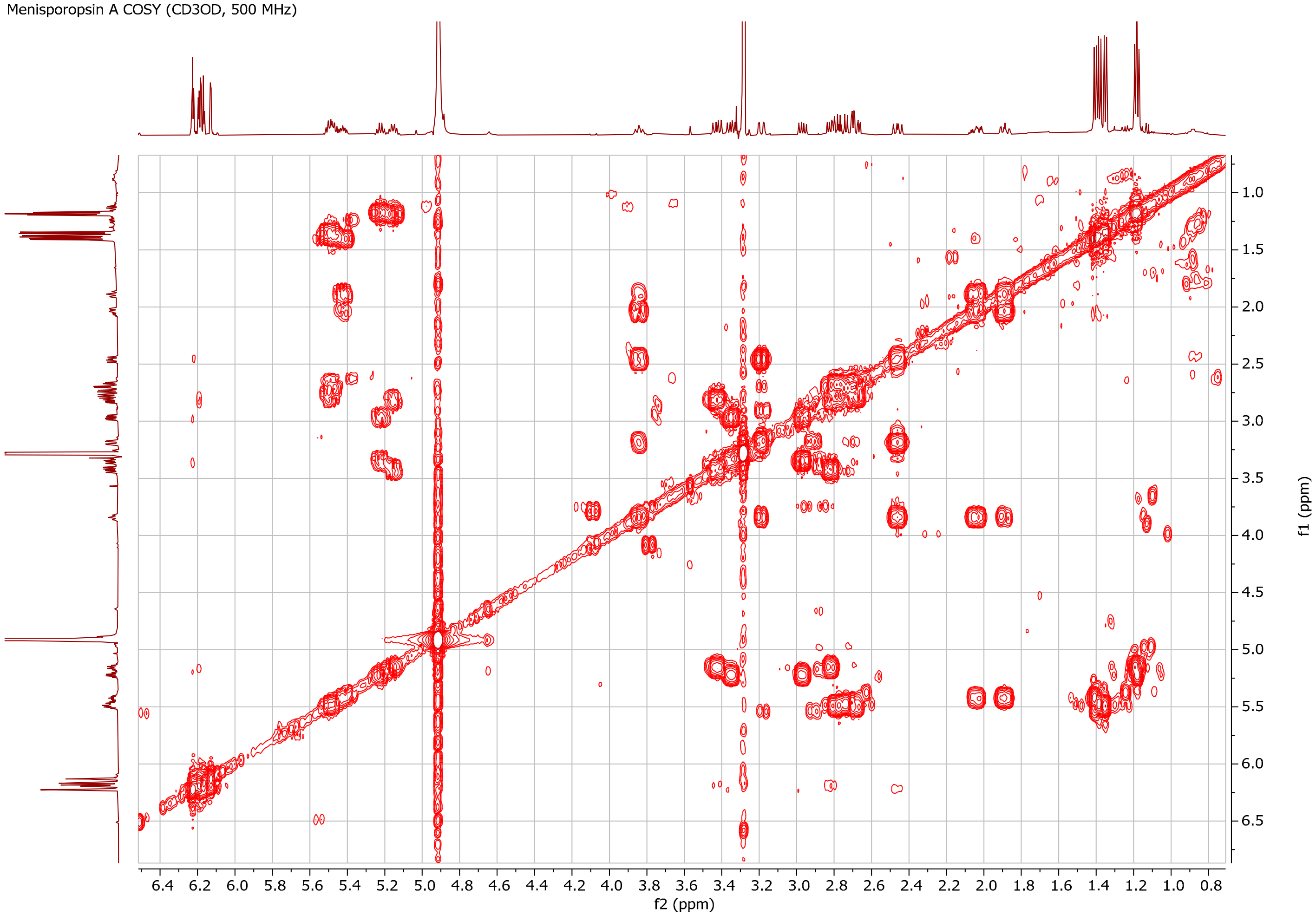

Figure S5. ^{1}H-^{1}H (J^3) COSY spectra of menisporopsin A at 500 MHz.

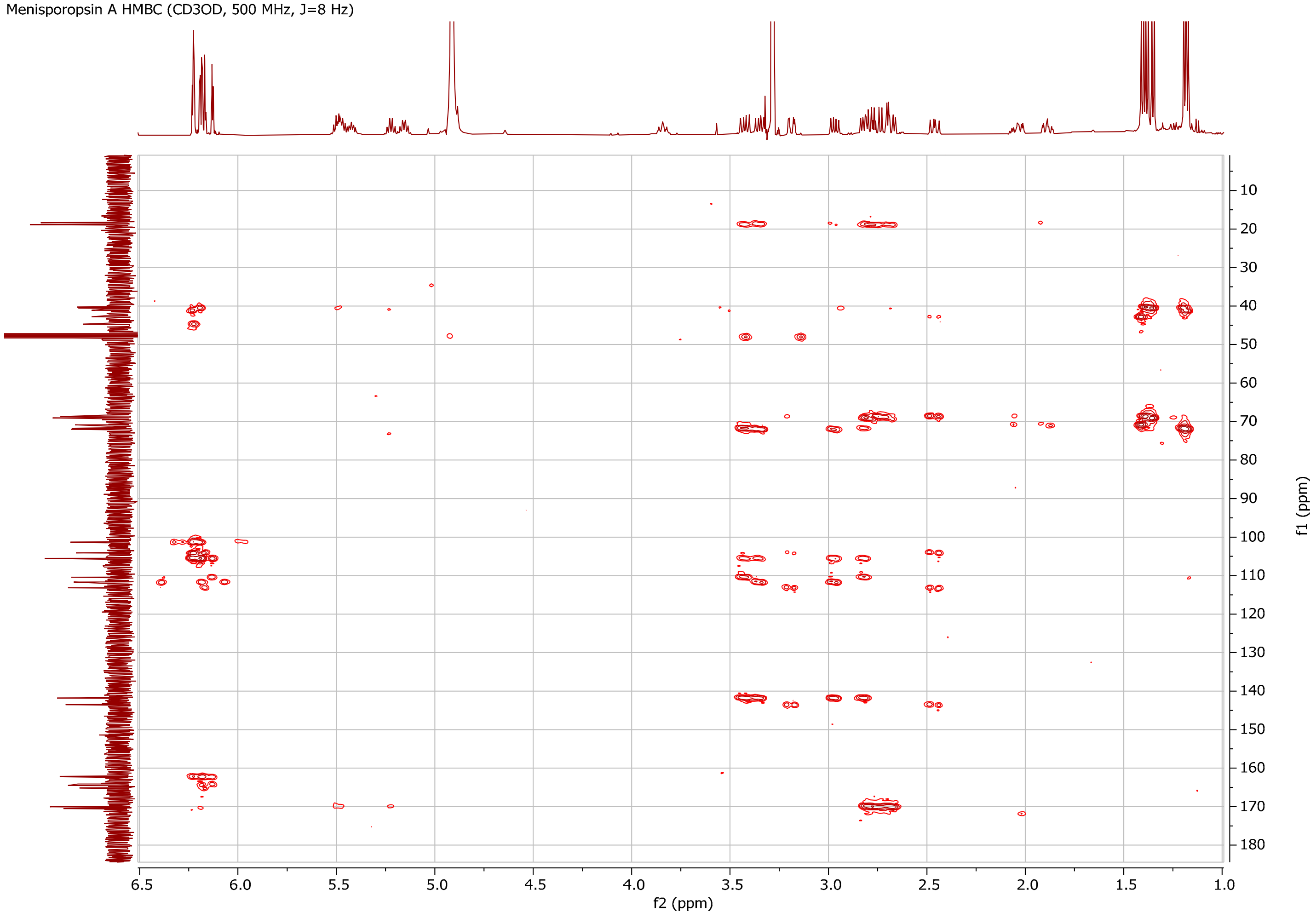

Figure S6. ^{1}H-^{13}C ($J^{3,4}$) HMBC spectra of menisporopsin A at 500 MHz.

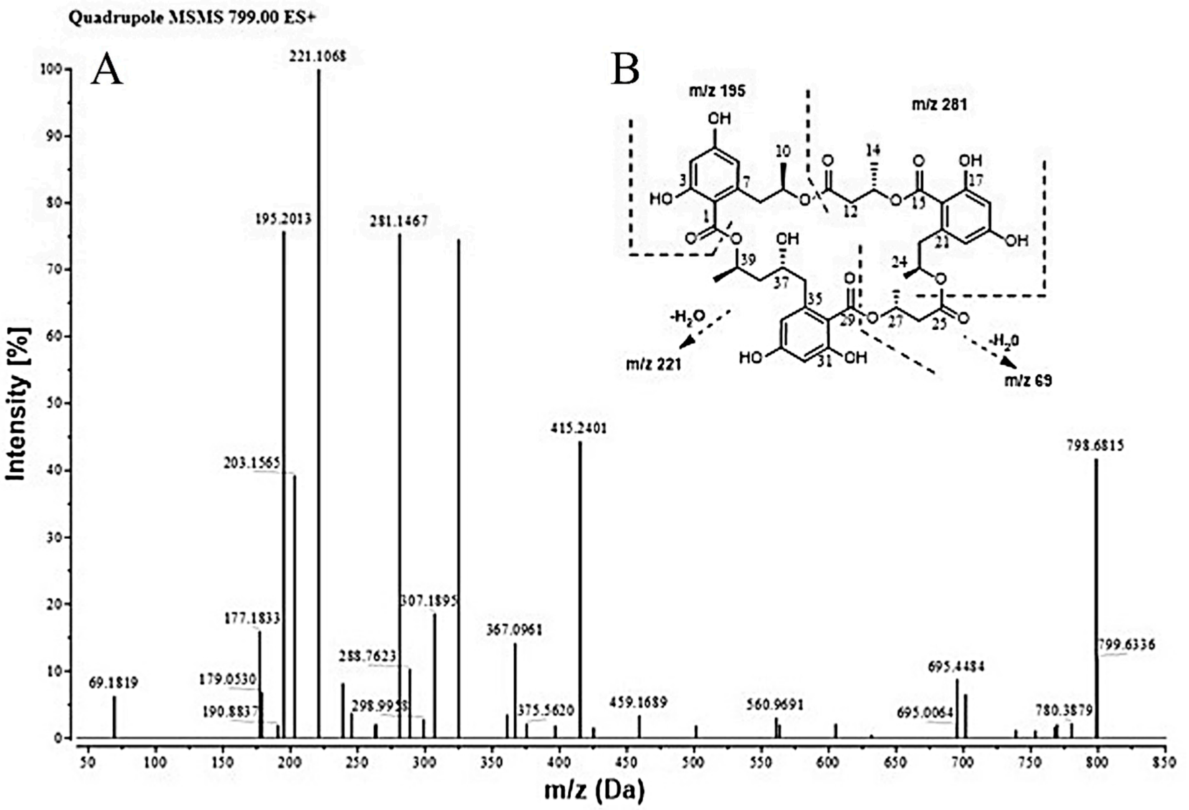

Figure S7. MS/MS spectrum for the fragmentation of menisporopsin A at 20 V (**A**). Chemical structure and cleavage sites for fragmentation of menisporopsin A (**B**).

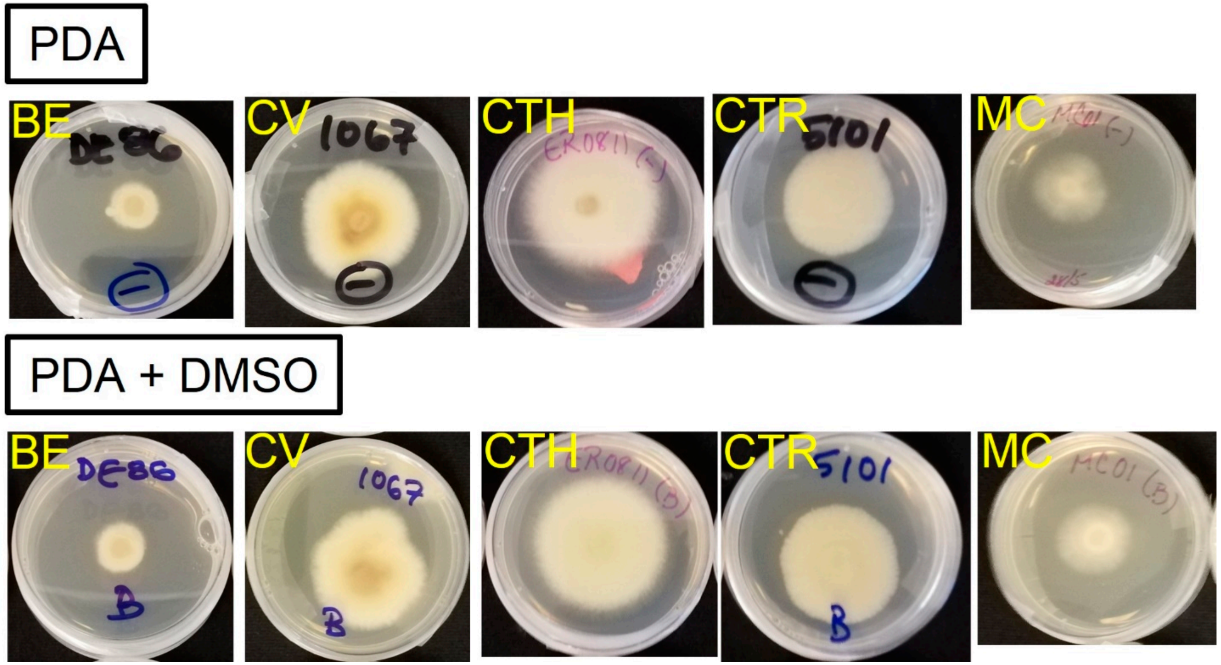

Figure S8. Growth of plant pathogens by the poisoned food method after 3 days at 25 °C on petri dishes containing potato dextrose agar (upper panel) and potato dextrose agar mixed with Dimethyl-sulfoxide at a final concentration of 0.5 % V/V (lower panel). Plant pathogens are coded as **BE** (*Boeremia exigua*), **CV** (*Calonectria variabilis*), **CTH** (*Colletotrichum theobromae*), **CTR** (*Colletotrichum tropicale*) and **MC** (*Mycena cytricolor*).

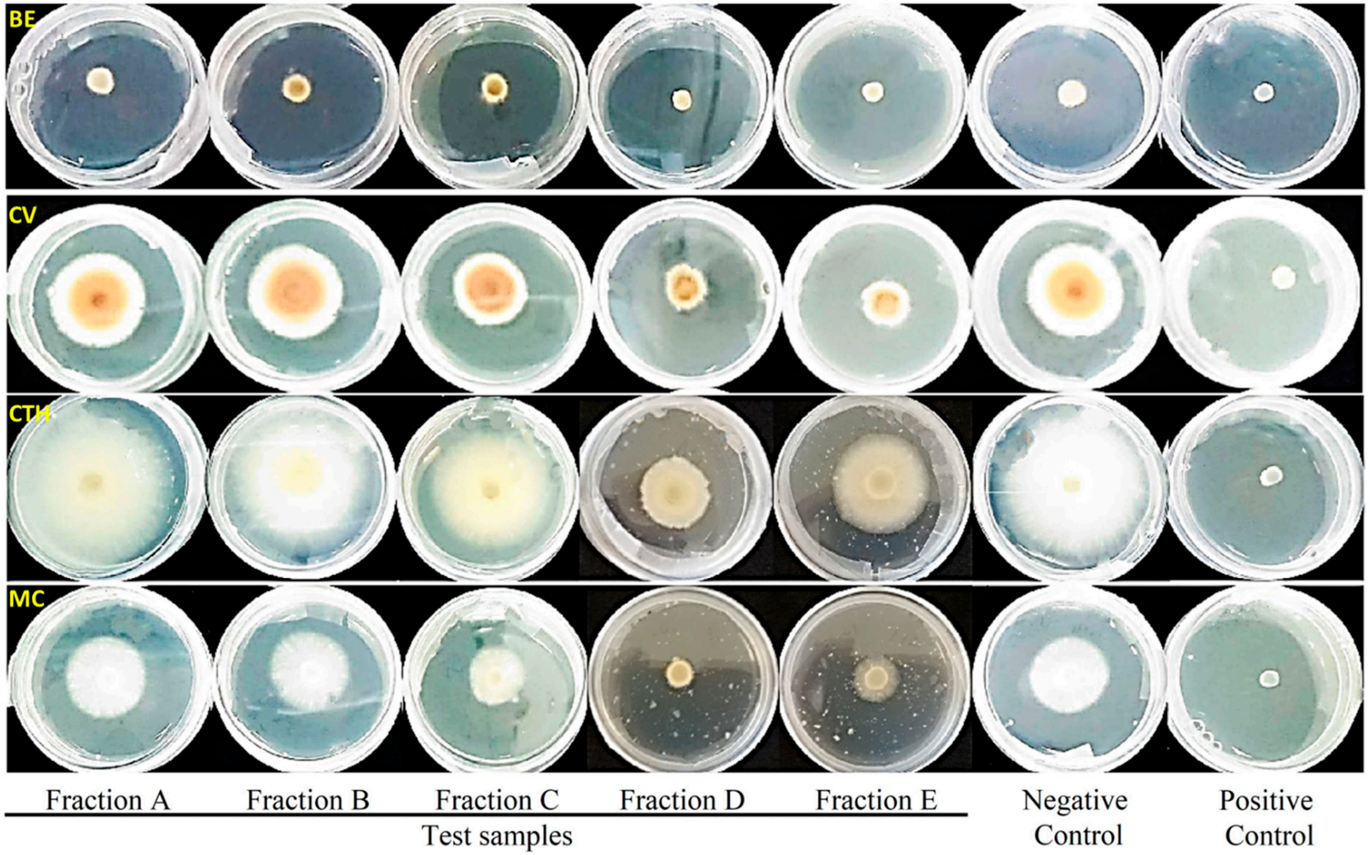

Figure S9. Antifungal activity against plant pathogens by the poisoned food method at 48 hours of column fractions from the endophytic fungus *Menisporopsis* sp. LCM 1078. Pathogens are coded as **BE** (*Boeremia exigua*), **CV** (*Calonectria variabilis*), **CTH** (*Colletotrichum theobromicola*) and **MC** (*Mycena citricolor*). Mancozeb (positive control) was evaluated at 50 μg/mL and DMSO at 0.5 %V/V was employed as the negative control. Growth inhibition was calculated in comparison with the negative control.

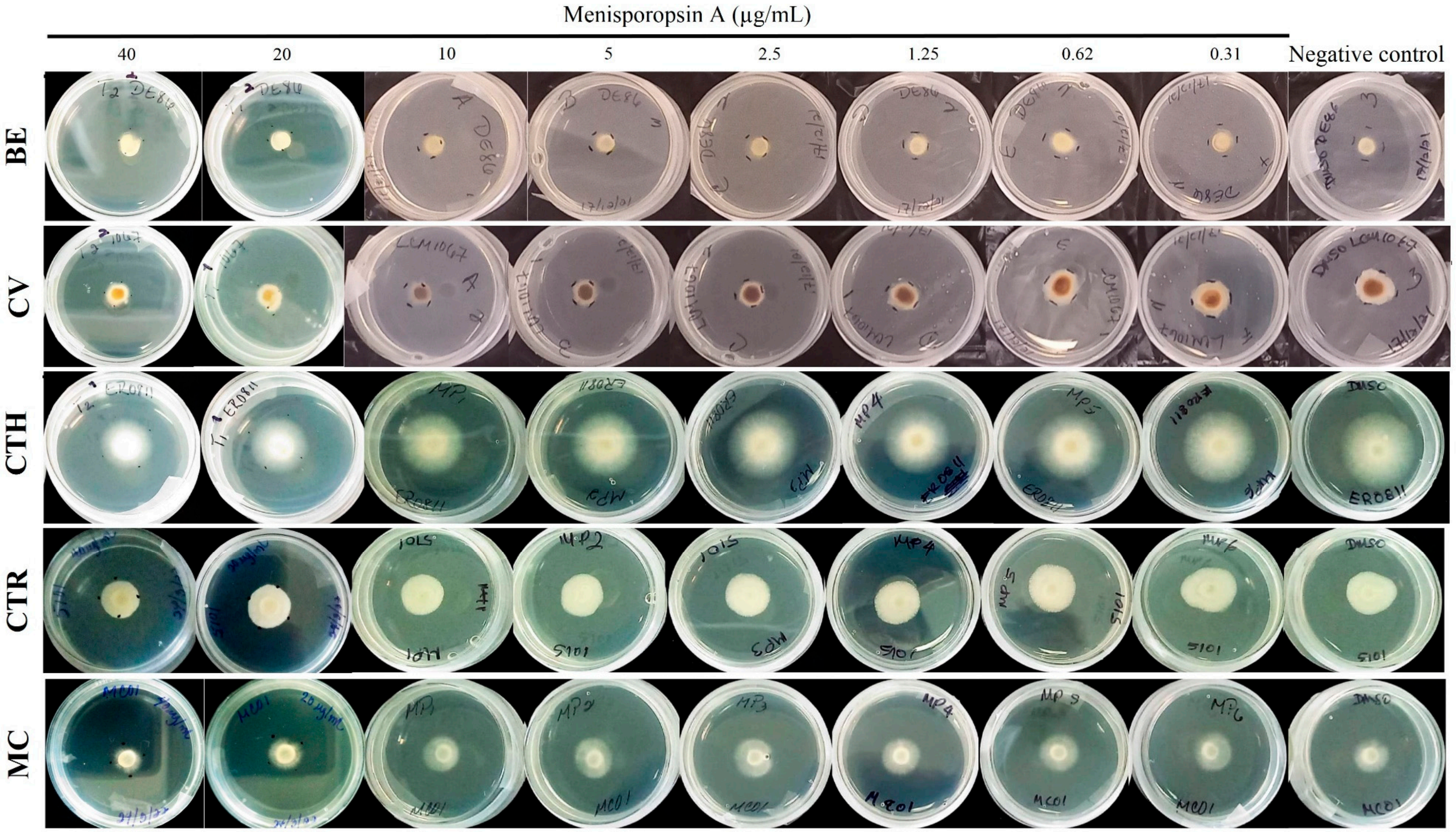

Figure S10. Antifungal activity against plant pathogens by the poisoned food method at 48 hours of menisporopsin A. DMSO at 0.5 %V/V was employed as the negative control. Growth inhibition was calculated in comparison with the negative control. Fungi are coded as **BE** (*Boeremia exigua*), **CV** (*Calonectria variabilis*), **CTH** (*Colletotrichum theobromicola*), **CTR** (*Colletotrichum tropicale)*, and **MC** (*Mycena citricolor*).

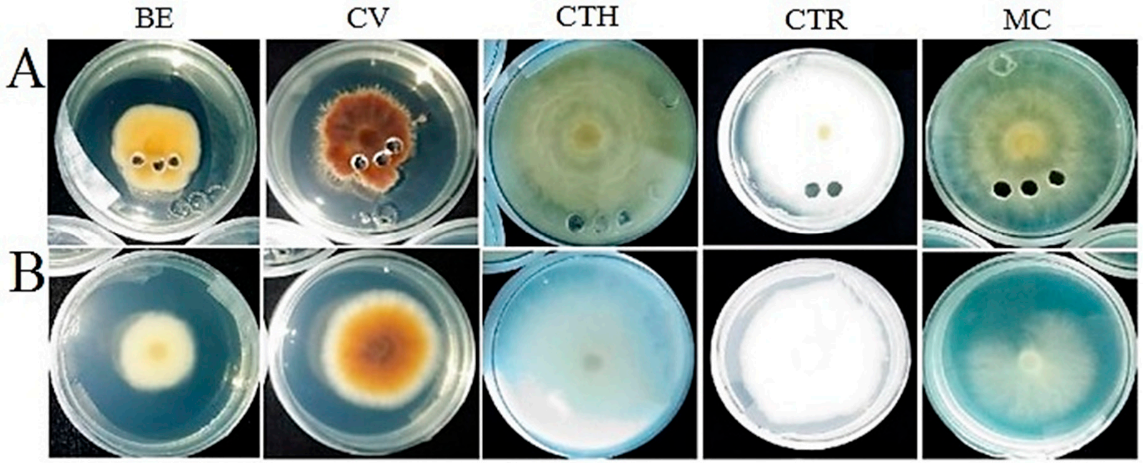

Figure S11. Treatment by the poisoned food method with menisporopsin A at 50 μg/mL by 7 days (**A**) and regrowth on PDA after 3 days. A plug taken from pathogen that were under treatment with menisporopsin A (**B**). Fungi are coded as **BE** (*Boeremia exigua*), **CV** (*Calonectria variabilis*), **CTH** (*Colletotrichum theobromicola*), **CTR** (*Colletotrichum tropicale)*, and **MC** (*Mycena citricolor*).

Table S1. ^{13}C NMR chemical shifts of menisporopsin A at 125 MHz.

Pos.[1]	δ in acetone-d		δ in methanol-d	Pos.[1]	δ in acetone-d		δ in methanol-d
	Menisporopsin A Literature [1]	Menisporopsin A Experimental	Menisporopsin A Experimental		Menisporopsin A Literature [1]	Menisporopsin A Experimental	Menisporopsin A Experimental
**1**	171.00	171.40	171.62	**21**	143.00	143.37	143.12
**2**	105.80	105.97	106.86	**22**	41.20	41.46	41.90
**3**	165.90	166.27	165.83	**23**	72.20	72.60	73.06
**4**	102.30	102.62	102.73	**24**	19.40	19.67	19.72
**5**	162.60	163.19	163.58	**25**	170.00	170.38	171.38
**6**	112.50	112.98	113.00	**26**	40.70	41.05	41.62
**7**	143.00	143.42	143.14	**27**	69.30	69.66	70.03
**8**	41.60	41.98	42.45	**28**	19.60	19.98	20.14
**9**	72.40	72.72	73.33	**29**	171.20	171.59	171.84
**10**	19.50	19.87	20.00	**30**	104.70	104.77	105.43
**11**	169.80	170.19	171.28	**31**	166.30	166.65	166.48
**12**	40.90	41.36	41.80	**32**	102.00	102.26	102.48
**13**	69.80	70.13	70.41	**33**	162.90	163.45	163.61
**14**	19.70	20.08	20.21	**34**	113.90	114.49	114.51
**15**	171.30	171.74	171.86	**35**	144.80	145.20	144.86
**16**	105.50	105.69	106.86	**36**	45.60	46.03	46.03
**17**	165.70	166.02	165.47	**37**	69.10	69.41	69.97
**18**	102.20	102.57	102.61	**38**	43.60	43.87	44.14
**19**	162.60	163.13	163.47	**39**	71.70	72.05	72.18
**20**	111.60	112.00	111.75	**40**	19.60	20.0	20.12

[1]Assignments were done by analysis of DEPT-135, DEPT-90, COSY, HSQC and HMBC NMR spectra.

[1]: Chinworrungsee, M.; Kittakoop, P.; Isaka, M.; Maithip, P.; Supothina, S.; Thebtaranonth, Y. 2004. Isolation and Structure Elucidation of a Novel Antimalarial Macrocyclic Polylactone, Menisporopsin A, from the Fungus *Menisporopsis Theobromae*. *J. Nat. Prod.* **67**, 689–692.
